# Recycling and Reprocessing of Thermoplastic Polyurethane Materials towards Nonwoven Processing

**DOI:** 10.3390/polym12091917

**Published:** 2020-08-25

**Authors:** Bastian Wölfel, Andreas Seefried, Vincent Allen, Joachim Kaschta, Christopher Holmes, Dirk W. Schubert

**Affiliations:** 1 Institute of Polymer Materials, Friedrich-Alexander University, Erlangen-Nürnberg (FAU), Martensstraße 7, 91058 Erlangen, Germany; vincent.allen@fau.de (V.A.); joachim.kaschta@fau.de (J.K.); 2 Adidas AG, Adi-Dassler-Straße 1, 91074 Herzogenaurach, Germany; andreas.seefried@adidas.com (A.S.); chris.holmes@adidas.com (C.H.)

**Keywords:** recycling, reprocessing, TPU, thermoplastic elastomers, TPE, thermoplastic, polyurethane, elastomers, nonwoven, processing, melt blown, molar mass, mechanical properties, methylendiphenylisocyanate, MDI, polytetramethylene glycol, PTMG, morphology, structure, phase separation, sustainability, circular economy

## Abstract

Thermoplastic Polyurethane (TPU) is a unique tailorable material due to the interactions of hard and soft segments within the block-copolymer chain. Therefore, various products can be created out of this material. A general trend towards a circular economy with regards to sustainability in combination with TPU being comparably expensive is of high interest to recycle production as well as post-consumer wastes. A systematic study investigating the property changes of TPU is provided, focusing on two major aspects. The first aspect focuses on characterizing the change of basic raw material properties through recycling. Gel permeation chromatography (GPC) and processing load during extrusion indicate a decrease in molar mass and consequently viscosity with an increasing number of recycling cycles. This leads to a change in morphology at lower molar mass, characterized by differential scanning calorimetry (DSC) and visualized by atomic force microscope (AFM). The change in molar mass and morphology with increasing number of recycling cycles has an impact on the material performance under tensile stress. The second aspect describes processing of the recycled TPU to nonwoven fabrics utilizing melt blowing, which are evaluated with respect to relevant mechanical properties and related to molecular characteristics. The molar mass turns out to be the governing factor regarding mechanical performance and processing conditions for melt blown products.

## 1. Introduction

Thermoplastic Polyurethane (TPU) belongs to the class of thermoplastic elastomers, combining desirable elastomeric properties at application temperatures and thermoplastic processability. Their characteristics are based on the intra- and intermolecular interactions in the block-copolymer chain consisting of hard and soft segments. The possibility of creating different soft-to-hard-block-ratios makes it a unique tailorable material and implies the main advantage over classic homo-polymers. Consequently, the application range comprises e.g., biomedical products, filtration, furniture, construction materials, insulation, and footwear amongst others, making polyurethane products the fifth-biggest polymer market globally [1,2,3,4,5,6,7]. With TPUs being comparably expensive, it is of high interest to recycle production wastes like trim-cuts or gating systems as well as post-consumer waste.

The block-copolymer chain of TPU typically consist of an isocyanate hard segment and ester or ether based soft segmentsHad. Examples for commercially applied hard segment base constituents are hexamethylene diisocyanate (HDI) [8], toluene diisocyanate (TDI) [9,10,11] and methylendiphenylisocyanate (MDI) [12,13,14,15,16,17,18,19], combined with soft segments like polyethylene glycol (PEG) [20,21] as an example for ester based or the ether based polytetrahydrofurans (PTMEG/PTMG) [8,18,19,22]. As a result of the stronger affinity of the hard segments to the ester groups than to the ether groups in the soft segment, the solubility of the hard segments in the ester elastomer is higher and their segregation lower than in the ether elastomer [11,23,24]. Accordingly, the properties for ester- and ether-based materials exhibit different peculiarities, with the latter named type comprising advantageous performances in terms of flexibility, especially at low temperatures, hydrolysis, and resistance to microbes. The last two factors are of particular importance regarding filtration applications, where melt blowing is state of the art [25]. Commercially applied materials in industry, as well as in sports engineering, are ether-based TPUs consisting of a MDI+BD (butane diol) hard and PTMG (polytetramethylene glycol) soft segment (SS) [7,22].

**Figure 1:**
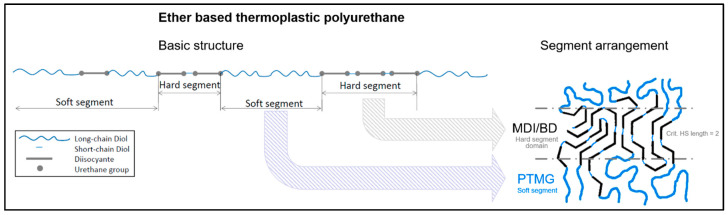
Schematic example of the arrangement of ether based thermoplastic polyurethane consisting of methylendiphenylisocyanate (MDI)/BD hard and PTMG soft segments after [13,17,18,26].

To form hard segment (HS) domains, the hard and soft segments (SS) in the chains must separate into different phases on the micro level. The arrangement into hard segment domains can only be successful above a critical hard segment length as displayed in Figure 1. Below this, the hard segments are dissolved in the soft phase and can therefore not function as a hard segment [13,17,18,26].

## 2. Motivation

Recycling TPUs has been named in several patents describing a supplemental option for a nonwoven fabrication process, but was neither specified further, nor published. All scientific relevant studies concerning the recycling of TPUs target injection molded specimens in comparison to extrusion. Degradation occurs in all kinds of thermoplastic processes and can interfere with the crystallization processes. This is especially distinct during injection molding, where varying orientation and cooling conditions influence the resulting measured mechanical properties.

A systematic study investigating the property changes of reprocessed TPU from a raw material perspective is provided. It is well known that during (multiple) processing the molar mass decreases; this was quantified by gel permeation chromatography (GPC). The mechanical properties of polymers depend on their molecular structure as Flory has already shown in 1945 [27]. Therefore, mechanical tests on plates of controlled equal pressing conditions have been conducted with regard to tensile stress and elongation at break. The change in molar mass with an increasing number of cycles additionally has an influence on the crystallization behavior of the TPU [12,26,28], but has, until now, never been related to an actual recycling study.

Subsequent processing of the recycled TPUs to nonwoven fabrics using melt blowing is also evaluated and the fabric properties are related to molecular characteristics. The molar mass is the governing factor regarding mechanical performance and processing conditions for melt blowing. This has been characterized through the examination of the obtained nonwovens via tensile and tear strength, as well as GPC.

A major characteristic of TPUs is that the onset of degradation mechanisms is already within the recommended processing window provided by manufacturers of commercial products [8,15,18,19,21,26,29,30]. Therefore, it is of enormous importance to identify the correlation between (multiple) processing, the changed molar mass and resulting structures.

## 3. Materials and Methods 

**Figure 2:**
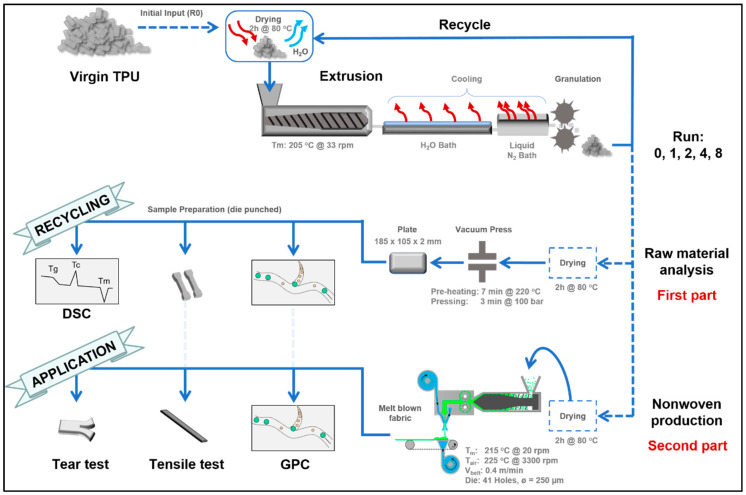
Schematic of the experimental setup: Reprocessing of the Thermoplastic Polyurethane (TPU) and characterization of the impact of recycling on the bulk material and application of the recycled materials as nonwoven through melt blowing. Relevant processing and sample preparation parameters are indicated.

The experimental setup displayed in Figure 2 is comprised of three major steps: material compounding, raw material analysis in the context of recycling, and applying these materials in nonwoven production.

A polyether-based thermoplastic polyurethane resin of 80A Shore hardness, consisting of MDI+BD hard and PTMG soft segments and featuring a hard segment content of roughly 30 wt% was used in the study. Prior to compounding the TPU material was dried for two hours at 80 °C in a Motan (Motan Holding GmbH, Konstanz, Germany) Luxor dehumidifier before every processing step and is therefore considered the standard drying procedure. Compounding was performed in a Leistritz (Leistritz Extrusionstechnik GmbH, Nürnberg, Germany) twin screw extruder with kneading elements at 205 °C and 33 rpm, followed by a two-step cooling procedure using water and liquid nitrogen to allow for granulation. The granulated material was then reprocessed using the same steps a total of 8 times. Material was retained at completed processing of runs 1, 2, 4, and 8 to perform both the raw material analysis, part one, and nonwoven production, part two. Included in the results is a comparison to the virgin raw material, which did not undergo the extrusion process, i.e., Run 0. The chosen processing temperature is based on the recommended processing windows provided by manufacturers of comparable grades and hardness. It ensures that all existent crystalline structures are transformed into molten state according to [17,26].

The first part deals with the material’s inherent change due to the recycling procedure. Hence the material was dried, pressed into plates using a Vogt (VOGT Labormaschinen GmbH, Berlin, Germany) vacuum press and analyzed regarding change of molar mass, mechanical behavior, and material structure. The samples for mechanical analysis were punched out from the bulk material plates in dimensions comparable to DIN ISO 527-2 1BB mini dog-bones. For determining material structure, differential scanning calorimetry (DSC) Q2000 from TA Instruments Inc. (New Castle, DE, USA) was used with a heating rate of 10 °C/min in a range of 20 to 240 °C and sample size of 6–8 mg was performed.

The second part compromises the processing of the before characterized recycled materials into nonwovens. Therefore, a melt blowing line from FET (Fibre Extrusion Technology Ltd., Leeds, UK) with a 14 cm wide die-head, comprising 41 holes with a diameter of 250 µm, was used. The melt temperature was 215 °C. A throughput of 0.007 cm^3^/s per hole was achieved by the melt pump at 20 rpm. Hot air (ot air225 °C, 1830 L/min) was blowing the melt onto the 0.4 m/min travelling belt. The resulting roughly 15 cm wide nonwoven was analyzed in terms of resulting molar mass and its mechanical behavior regarding tensile strength in machine direction (MD) and cross direction (CD) and tear strength in MD. The specimens for tensile testing, sized as a rectangle of 10 mm × 90 mm, were punched out of the nonwoven. The tear strength samples are adapted to DIN ISO 34-1 and rescaled to 35 mm × 70 mm with a 35 mm incision in MD justified by the nonwoven sizing. A Zwick-Roell Z050 tensile testing machine (ZwickRoell GmbH & Co. KG, Ulm, Germany), with a 1 kN load cell and pneumatic clamps under 7 bar pressure, performed the mechanical analysis.

All Differences in molar mass were characterized by gel permeation chromatography (GPC 1260 Infinity II, Agilent Technologies, Santa Clara, CA, USA) with *N*,*N*–Dimethylacetamid (DMAc) and Lithium-Bromide (LiBr) as a solvent. The used columns (set to 60 °C) are supplied by PSS (PSS Polymer Standards Service GmbH, Mainz, Germany) and combined with a dRI detector Shodex 101, LS detector Wyatt Minidawn (Wyatt Technology Corporation, Santa Barbara, CA, USA). The flow rate of the mobile phase was 1 mL/min. The samples were measured and evaluated using the software “PSS SECurity^2^ GPC System” (Build 8251, PSS Polymer Standards Service GmbH, Mainz, Germany).

## 4. Results and Discussion

**Figure 3:**
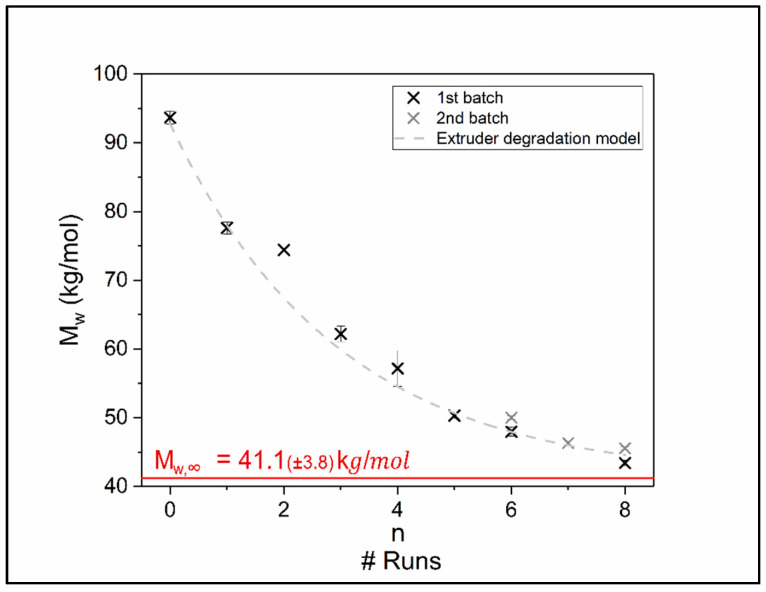
Decrease of *M*_w_ as a function of recycling runs for fixed temperature (T = 205 °C) and fixed extruder rotational speed (33 rpm). The dashed line shows the best fit of Equation (1).

Figure 3 displays the change of chain length expressed by the weight average molar mass *M*_w_ as a function of the number of recycling runs. It is observed that the raw material reveals an asymptotic decrease in molar mass when reprocessed in an extruder. The molar mass is not dropping under roughly 40 kg/mol when reaching an equilibrium state, which may be due to concurrent degradation and build up processes in the TPU which have been described in literature [18].

As the degradation mechanisms are of mechanical and thermal nature resulting in a decay of molar mass (*M*_w_) over recycling steps (*n*), this context can be described by an exponential decay function:

(1a) $$\begin{array}{cc} & M_{w}(n)=(M_{w,0}-M_{w,\infty })*\exp(-n/ñ)+M_{w,\infty }\\ \text{with}: & M_{w,0}=\left(92.7\pm 2.3\right)\;\mathrm{kg}/\mathrm{mol}\\ & M_{w,\infty }=\left(41.1\pm 3.8\right)\;\mathrm{kg}/\mathrm{mol}\\ & ñ=3.0\pm 0.5 \end{array}$$

In this relation *M*_w,0_ describes the initial molar mass of the virgin product, *M*_w,∞_ is the asymptotic molar mass after an infinite number recycling steps and *ñ* describes the degree of decay per recycling run in molar mass degradation. Equation (1a) is attributed to the solution of the first order differential equation:

d*M*_w_/d*n* = −(*M*_w_ − *M*_w,∞_)/*ñ* (1b)

Beside the temperature, the degradation is furthermore depending on the mechanical forces transmitted onto the polymer chain. As the shear forces are dependent on viscosity and thus on chain length it is obvious that the forces applied to individual chains have to decrease with increasing number of recycling runs.

**Figure 4:**
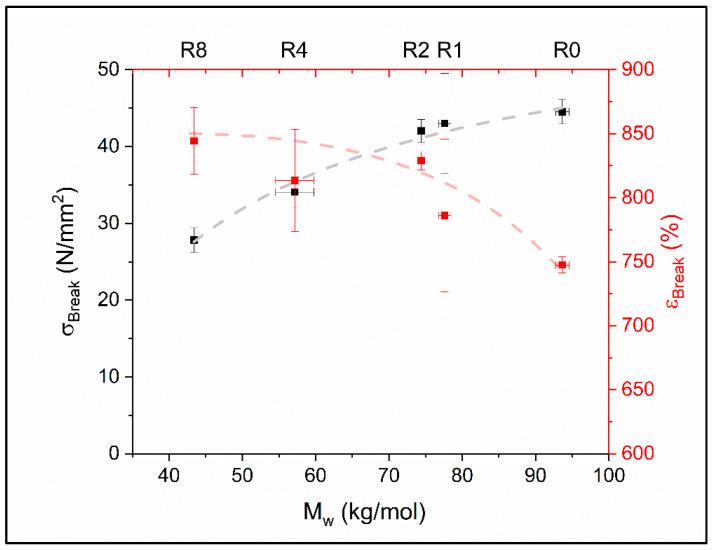
Stress and elongation at break of recycled TPU as a function of weight average molar mass *M*_w_. The top axis indicates the recycling step Rx (x = 0, 1, 2, 4, and 8). The red dashed line is a guide to the eye whilst the grey line displays the best fit of Equation (2).

A rather surprising behavior of the bulk material can be observed in the tensile tests, displayed in Figure 4. While the stress at break decreases with reprocessing (decreasing *M*_w_) according to Flory’s [27] suggestion, the elongation at break increases. It is worth to note that most commonly used materials become brittle as a result of the decreasing chain length due to recycling. This inevitably leads to the finding that the material becomes less strong and more ductile. The dependence of the stress at break on *M*_w_ can be described by Flory’s [27] equation as:

(2) $$\begin{array}{cc} & \sigma_{\text{Break}}(M_{w})=A-B/M_{w}\\ \text{with}: & A=\left(60.0\pm 1.6\right)\;N/{\mathrm{mm}}^{2}\\ & B=\left(1400\pm 100\right)\;\left(N/{\mathrm{mm}}^{2}\times \mathrm{kg}/\mathrm{mol}\right) \end{array}$$

The values suggest that the stress at break could not exceed 60 N/mm^2^ even for infinitely high molar masses. However, it is very unlikely to obtain TPU products of this composition exceeding the framework of the investigated molar masses. The virgin product and the eight times recycled material state the entire possible molar mass values, making it an interpolative view and awarding additional interpretive strength to this relation. The elongation behavior is not further described based on this plot, as it does not follow common rules and requires a separate follow up discussion.

**Figure 5:**
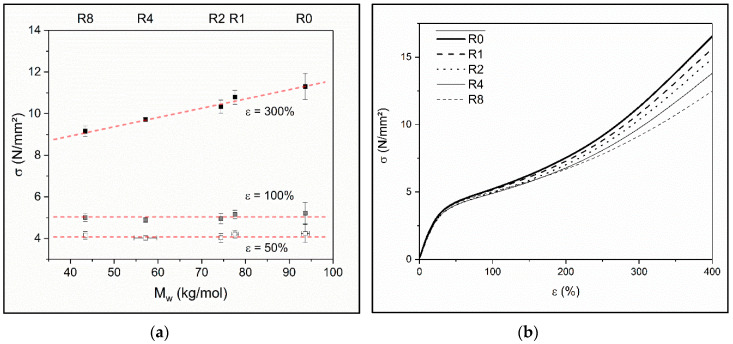
(**a**) Tensile stress of recycled TPU material for different elongations as a function of weight average molar mass *M*_w_. The top axis indicates the recycling step Rx (x = 0, 1, 2, 4, and 8); (**b**) Corresponding mean stress–strain–curves of the measured values from recycling step Rx (x = 0, 1, 2, 4, and 8) shown in (**a**).

Considering the display of the stress at different elongations in Figure 5a and the slope of corresponding stress-strain-curves in Figure 5b, the presumption of a gain in ductility can be assumed confirmed. However, this effect is only evident for deformations larger than 100% and hence raises the question if there is a modification of the hard segments taking place during recycling and which parts of the TPU degrade.

**Figure 6:**
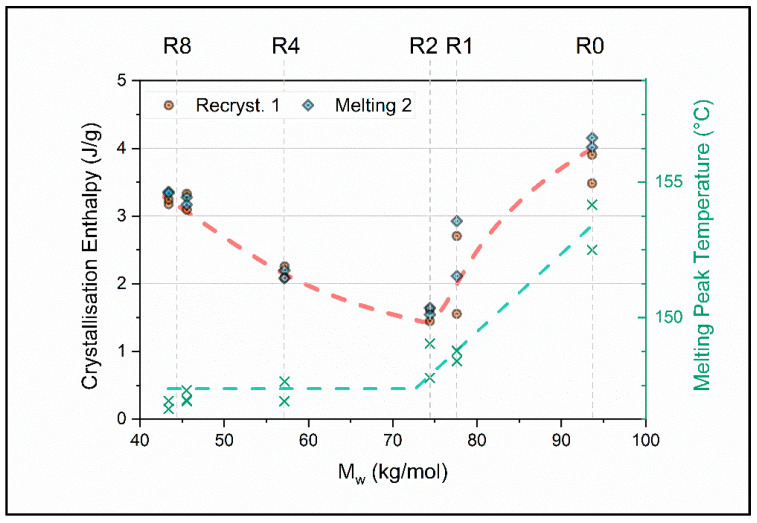
Left axis: Enthalpy of crystallization as a function of weight average molar mass *M*_w_. Right axis: Corresponding melting peak temperature in dependence of the average molar mass. The top axis indicates the recycling step Rx (x = 0, 1, 2, 4, and 8). The dashed lines are a guide to the eye.

**Figure A1:**
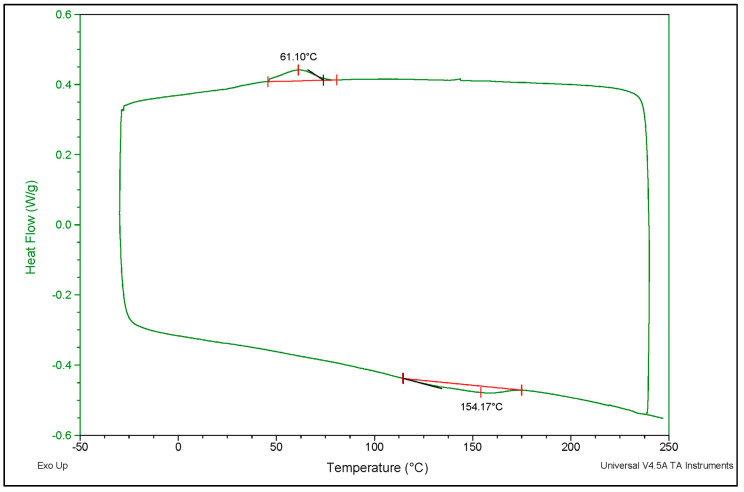
Exemplary differential scanning calorimetry (DSC)-curve of the first cooling and second heating run of the virgin (R0) material under 10 K/min temperature ramp. The corresponding crystallization and melting peaks are highlighted.

To get to the reasons for the peculiar behavior, DSC measurements were performed and the enthalpy of crystallization during cooling after the first heat up are compared and benchmarked to their melting enthalpy in the following heating run number two (Figure 6). An exemplary DSC measurement can be understood in Figure A1 in the Appendix A. The melting enthalpies are effectively identical to their enthalpies of crystallization, which assures we are not measuring an artefact. From Figure 6 it can be observed that crystallinity, represented by the enthalpy of crystallization, is decreasing from the virgin material towards the two times recycled material by about 60%. From the highly amorphous material obtained in run two, crystallinity rises again with the following recycling steps, almost to the initial value of the virgin material. This leads to the hypothesis that there are two separate degradation regimes for soft and hard segments of which the TPU consists. As TGA analysis of comparably chemically structured TPUs have shown [14,15], the hard segments start to degrade at around 200 °C, whereas the soft segments only degrade at temperatures above 300 °C. Therefore, the HS will degrade at processing temperature while the SS remain stable [14,15]. Hence after the degradation of the hard segments, it is assumed that the soft segments that constitute the governing resistance to melt flow and are, therefore, degraded from run two onwards.

This is backed by the corresponding peak temperature of the melt peak in the second heating run. It can be observed that after the second recycling step the values are constant. The behavior of the peak temperature in correspondence to the present hard domain structure was analyzed in detail by Hu and Koberstein et al. and Martin et al. and published in 1994 and 1997, respectively [17,26]. They found that a decrease in average hard segment length directly leads to a lower melting peak temperature. This results from the higher order of hard segments that can be achieved upon a higher number of H-bonds inside the hard phase. This makes the structure more resistant to phase transformation. Based on this it can be concluded that the hard segment structure remains constant for recycling runs greater than two and therefore any changes in crystallinity after recycle run 2 are based on changes in the soft segments. The general molecular structure of this MDI/BD TPU is not changing during processing as it is subject to “transurethanisation” [17,18,29,31]. The dissociation of the urethane bond to free isocyanate and hydroxyl end-groups is the primary degradation mechanism: From a temperature between 200 until 330 °C isocyanates and alcohols are the exclusively appearing degradation products [15,29,30]. 

**Figure 7:**
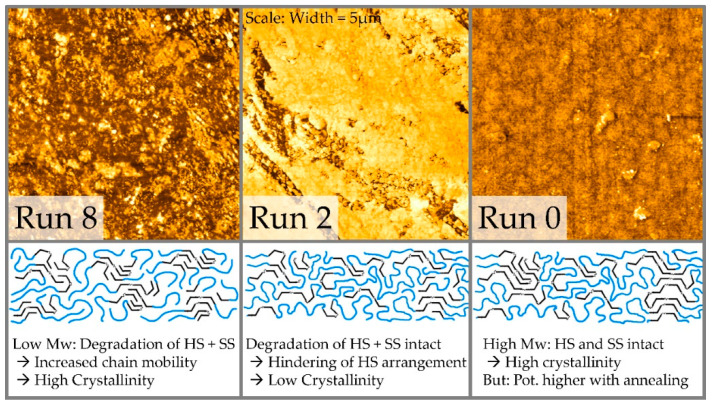
AFM pictures of the morphology of virgin (R0), two times recycled (R2) and 8 times recycled (R8) TPU together with possible hard segment (HS)- and soft segment (SS)-structures. The sides of each image have a length of 5 µm.

Attempting to obtain further evidence for the hypothesis of two regimes, AFM images of the samples were recorded and the phase images are displayed in Figure 7.

It can be observed that finely structured and clearly separated, discrete phases of different resistance to the cantilever are present in the Run 8 material. Constituted of short chains, which feature an increased mobility, the shortened HS are likely to separate and form crystals. In the Run 2 material, the SS are still intact and likely to hinder the arrangement of already shortened HS, leading to a low crystallinity. This can be seen in the AFM image with a high amount of bright SS phase with isolated darker HS phase. In the virgin R0 material both phases are assumed to be intact and well intermixed, wherefore no clear separation within scale is detectable. Long chains need more time to diffuse and phase separate, thus for the virgin material potentially higher crystallinity could be achieved by applying an additional annealing procedure [26].

The conclusion from the first part targeting the raw material properties is that the present TPU degrades in two steps. As the recommended processing temperatures of the TPUs are above 200 °C and therefore already inside the degradation regime of the hard segments, these are the first ones to degrade. After two steps of recycling in the extruder the hard segments are already degraded to a large extent. The shortage of hard segments is reflected by a decrease in T_m_ during DSC. This is the reason why they cannot assemble to a proper hard phase anymore as they are sterically hindered by the intact soft segments. This is the reason why the AFM shows a large amount of bright soft phase. The soft segments are then degraded afterwards, as they are now the main contribution to viscosity through their molar mass. It can only be the soft segments shortening to achieve further decrease in molar mass as the melting peak temperature reflecting hard segment length does not change after the second recycling step. After both parts of the TPU, soft and hard segments, are degraded, there is also a low molar mass. This leads to increasingly mobile chains being able to assemble more efficiently, which reflects in a higher melting enthalpy and the clearly structured hard phase observed for “Run 8” material via AFM. All these findings are additionally reflected in the presented results obtained from tensile tests.

**Figure 8:**
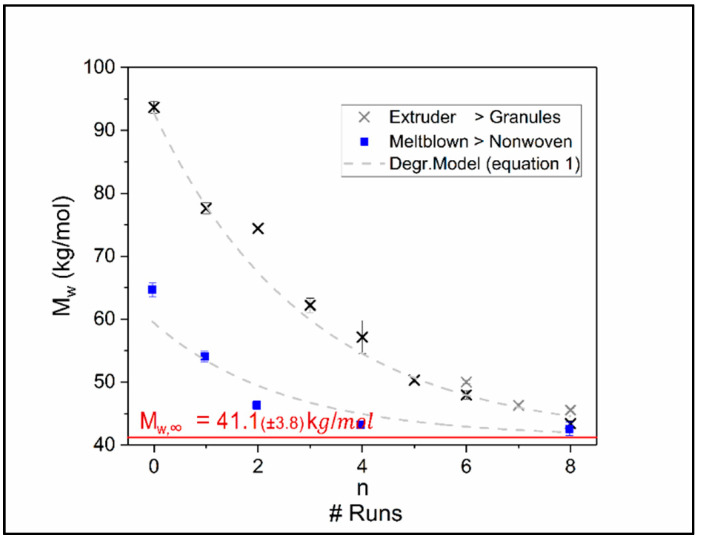
Decrease of *M*_w_ as a function of recycling runs for the material of the recycling study (crosses) and the nonwoven made out of it (rectangles). The dashed lines are the best adjustment of Equation (1).

The above described and analyzed recycled TPUs were processed to nonwoven fabrics using melt blowing. Figure 8 compares the *M*_w_-values of the fabrics to the values of the materials not being processed into nonwoven. Due to the higher temperatures in the melt blowing process together with an order of magnitude higher shear stress due to the small capillaries in the spinneret, the recorded molar masses of the nonwovens are smaller compared to the materials they were made of. However, the molar mass still does not drop under the asymptote discovered through multiple extrusions described above. This leads to the suggestion that this asymptote is a material specific parameter rather than dependent on processing conditions and is described in Figure 8. Processing TPU via melt blowing provides an inherently higher degradation and hence roughly adds three recycling steps compared to extrusion.

Due to the fact that two different processes, melt blowing and extrusion, with different typical shear rates yield the same asymptotic molar mass value within the experimental error, it is evident that local stresses acting on the polymer chains are identical. When the chains are initially longer, the process with a higher shear rate (or elongation rate) yields the higher stress on the chain. This results in a faster chain scission, therefore lowering the viscosity due to the molar mass reduction and finally reducing the stress on the chain. This means an equilibrium is reached independent of the initial shear rate (or elongation rate). The only constraint, which must be fulfilled that different processes yield the same asymptotic molar mass, is that the initial stress on the chain is larger than the critical stress needed for chain scission. If only thermal degradation would be relevant, the right side of Equation (1b), the differential equation would only consist of a constant and no *M*_w_ dependence would result. For TPUs the initial *M*_w_ itself is also dependent on the applied temperature as discussed in [18]. Therefore, one can assume that a thermal driven equilibrium molar mass is reached very fast followed by a subsequent mechanically driven degradation as described above.

**Figure 9:**
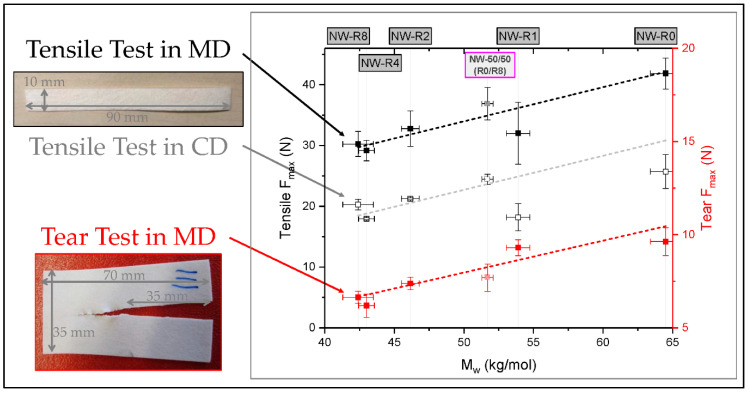
Tensile and tear strength of various nonwoven materials as a function of *M*_w_. The top axis indicates the starting material (NW-Rx) for the nonwoven (x = 0, 1, 2, 4, and 8). In addition a mixture of R0 + R8 (50%) is shown (its results are close to R1). The dashed lines are a guide to the eye.

As observed for the raw material, the mechanical performance of the nonwoven is dependent on the molar mass and displayed in Figure 9. Tensile strength in machine direction (MD), cross direction (CD) and tear strength (TS) follow a qualitatively comparable linear trend. This behavior is due to the formation of the nonwoven from single fibers. Generally, a higher number of fibers is orientated in MD, wherefore the tensile strength in this direction is expected to be higher. In this study the tensile performance in CD is roughly two-thirds of MD. The fiber orientation distribution, as well as the base weight of the fabric, play a significant role in the resulting mechanical properties. In nonwovens, the base weight corresponds to the sample thickness, in this study it is 305 g/m^2^ ± 5% for all samples. By keeping the processing conditions identical for all considered materials, the named above factors can be neglected for interpretation and resulting forces can be regarded as strengths. If tensile strength in MD and CD would not follow the same qualitative trend the learnings would be based on artefacts, e.g., incomparable fiber orientation distribution, and therefore represents a plausibility check. The only exception is the nonwoven material NW-R1. During its production die-hole blockages occurred for which reason these points shall be interpreted with caution.

**Figure 10:**
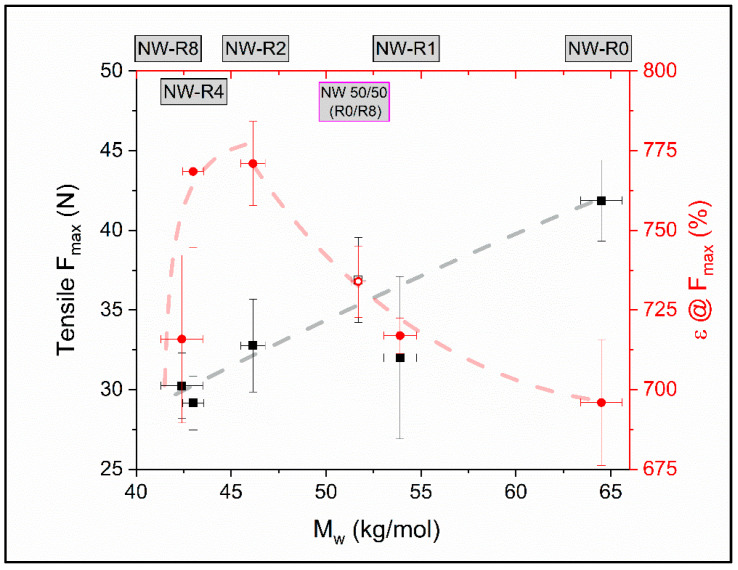
Force at break (*F*_max_) and elongation at break (ε@*F*_max_) as a function of *M*_w_ for various nonwovens (NW0-NW8, closed symbols) and a nonwoven made of 50/50 wt % mixture of R0 and R8 (open symbol). The dashed lines are a guide to the eye.

Comparing the maximum elongation at break to the respective force (Figure 10), it can be seen that it increases towards the fabric produced from twice recycled material. This can be explained by the already described properties of this material with regard to strongly pronounced soft phases and consequently lower crystallization and is visualized in Figure 10.

This observation is further indication towards the hypothesis of two separate degradation regimes for soft and hard segments. Producing nonwovens from 50/50 mixtures out of virgin and eight times recycled material, which is close to *M*_w,∞_, the resulting nonwoven properties are also mean. In the present case this equals a molar mass and the respective mechanical performance close to the fabric produced from once recycled material. This is a remarkable finding which holds the possibility to tailor the aspired material performance by mixing and opens the potential for products made from recycled TPU.

## 5. Summary and Conclusions

This study has revealed new insights into molar mass dependent TPU raw material behavior, as well as nonwoven attributes on an application relevant level. When reprocessed and therefore recycled, the raw material reveals an exponential decrease in molar mass with the number of recycling runs, but limited by an asymptote. It is not dropping under roughly 40 kg/mol when reaching an equilibrium state. A rather surprising behavior can be observed under tensile stress. While the stress at break is lower for smaller chain length and, therefore, follows Flory’s suggestion for molar mass dependence, the elongation at break meanwhile increases. The most commonly used materials become brittle as a result of recycling, this TPU gets weaker for deformations larger than 100% and stays identical within the errors for lower deformation. DSC measurements indicate that crystallinity of virgin and eight times recycled material (R8) is comparable. Whereas for two times recycled material, crystallization enthalpy decreases by about 60% approaching a minimum. This twice recycled material is highly amorphous in comparison due to hard segment (crystallinity creator) degradation. AFM images reveal different crystallization patterns for the various recycled materials. This leads to the hypothesis that there are two separate degradation regimes for soft and hard segments of which the TPU consists. Hard segments start to degrade at around 200 °C, which is inside the processing window of the material. Therefore, the HS degrades prior to the SS, whose thermal degradation does not start until 300 °C. Hence, after the degradation of the hard segments, it is assumed that the soft segments are responsible for resistance to melt flow and are, therefore, degraded from run two onwards.

Nonwoven fabrics using melt blowing were produced from the various recycled grades at constant extrusion conditions. Even though processing temperatures and shear rates have been one order of magnitude higher during melt blowing compared to the preceding extruder recycling, the molar mass still does not drop under the asymptote of 40 kg/mol discovered through multiple extrusion. This obvious independence of the asymptotic molar mass from temperature and shear conditions leads to the suggestion that this value describes a material specific parameter. The melt blowing process is roughly equivalent to 3 recycling steps which extends the study to effectively 11 recycling steps in total. As well as observed on raw material level, the mechanical performance of the nonwoven is dependent on the molar mass. Looking at the maximum elongation at break during tensile testing, it can be seen that it increases towards the fabric produced from material recycled twice and drops afterwards. This observation further supports the hypothesis of two separate degradation regimes for soft and hard segments. Producing nonwovens from 50/50 mixtures out of virgin and eight times recycled (close to the asymptotic molar mass) material results in nonwovens roughly featuring properties following a linear mixing rule with respect to molar mass dependence of properties. In the present case, this equals a molar mass and the respective mechanical performance close to the fabric produced from once recycled material. This is remarkable and holds the possibility to tailor the aspired material performance by mixing and opens up potential for tailoring products made from recycled materials.
